# P-668. Detection and Clinical Impact of Influenza in a Cohort of Agricultural Workers in Guatemala, 2020-2024

**DOI:** 10.1093/ofid/ofaf695.881

**Published:** 2026-01-11

**Authors:** Neudy C Rojop, diva M barrientos, Claire Bradley, Julio del Cid-Villatoro, Daniel Carreon, Ashley Fowlkes, Chelsea Iwamoto, Emily Zielinski-Gutierrez, Edwin J Asturias, Molly Lamb, Daniel Olson, Kareen Arias

**Affiliations:** Fundacion Para La Salud Integral de los Guatemaltecos, Los Encuentros, Retalhuleu, Guatemala; Fundacion para la Salud Integral de Los Guatemaltecos, Fraijanes, Guatemala, Guatemala; Fundación para la Salud Integral de los Guatemaltecos, Burlington, VT; Fundación para la Salud Integral de los Guatemaltecos, Burlington, VT; Centers for Disease Control and Prevention, Atlanta, Georgia; Centers for Disease Control and Prevention, Atlanta, Georgia; Center for Disease Control and Prevention, Atlanta, Georgia; Centers for Disease Control and Prevention Central America, Guatemala, Quetzaltenango, Guatemala; CU School of Medicine, Aurora, Colorado; Colorado School of Public Health, Aurora, Colorado; CU School of Medicine, Aurora, Colorado; Fundacion Para La Salud Integral de los guatemaltecos, Los Encuentros, Retalhuleu, Guatemala

## Abstract

**Background:**

Influenza viruses circulate year-round in Central America, but their impact on agricultural workers, essential to food security in the Americas, is poorly understood.

**Figure:**
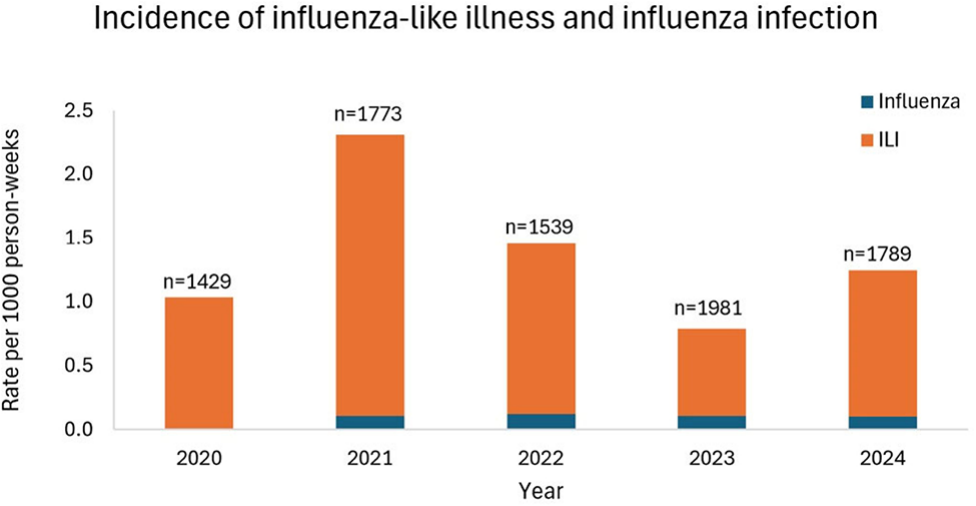

**Methods:**

We evaluated the incidence of influenza among a cohort of agricultural workers in southwest Guatemala participating in active surveillance for influenza-like illness (ILI) from June 2020 to October 2024. Workers reporting cough, fever, or shortness of breath in the previous 7 days provided nasopharyngeal swabs for influenza RT-PCR testing using the Roche cobas liat instrument. Symptom data were collected on the day of reporting, day 7, and day 28, with differences in reported symptoms by influenza virus infection status assessed using Fisher’s Exact tests.

**Results:**

Of 2,809 agricultural workers who participated in ≥1 year of surveillance, 76% were male with a mean age of 30 years. Overall, 400 (14%) ever reported an ILI episode; 7.3% (29/400) were influenza positive (flu+): 18 influenza A, 11 influenza B. The incidence of ILI and flu+ cases per 1000 person-weeks from 2021 to 2024 was 1.4 (95% Confidence Interval [CI] 1.3-1.6) and 0.11 (95% CI 0.07-0.16), respectively, with minimal yearly variation (Figure). Of the 400 ILI cases, 68% reported fever, 78% cough, and 28% shortness of breath. Flu+ cases reported fever more frequently than flu- cases (96.6% vs. 65.9%, p=0.001). Among ILI cases, 8.4% reported difficulty getting out of bed on day 7 and 3.4% on day 28, with no significant difference by influenza status.

**Conclusion:**

Among young adult, mostly male agricultural workers, 7.2% of reported ILI episodes were attributed to influenza, with fever being a key symptom. This study demonstrates the feasibility of monitoring circulating respiratory virus illnesses in field-based environments using active surveillance and rapid diagnostics.

**Disclosures:**

Edwin J. Asturias, MD, Pfizer: Grant/Research Support Molly Lamb, PhD, Merck: Grant/Research Support Daniel Olson, MD, Fundacion para la Salud Integral de los Guatemaltecos: Board Member|Merck: Grant/Research Support|Roche Diagnostics: Grant/Research Support

